# Leiomyoma treatment by uterine artery embolization using gelatin sponge prepared by the pumping method

**DOI:** 10.3892/etm.2012.688

**Published:** 2012-08-29

**Authors:** TAKAHISA KOJIMA, YASUNORI TAKI, HIDEFUMI FUJISAWA, KUMIKO KOYAMA

**Affiliations:** 1 Amor Clinic, Kanagawa 222-0033;; 2 Department of Radiology, Fuchu Keijinkai Hospital, Tokyo 183-0034;; 3 Department of Radiology, Showa University Northern Yokohama Hospital, Kanagawa 224-8503, Japan

**Keywords:** leiomyoma, uterine artery embolization, gelatin sponge, pumping method

## Abstract

Uterine leiomyoma, a benign tumor, may be treated with drugs, albeit surgical resection is more common. The present study aimed to evaluate the treatment of leiomyoma cases by uterine artery embolization (UAE) using a gelatin sponge prepared by the pumping method. The results of 700 leiomyoma cases treated by UAE in the outpatient department were evaluated. UAE was performed by injecting gelatin sponge prepared by the pumping method into the uterine artery. Among 700 cases, effective cases were 680 (97.2%), ineffective cases were 18 (2.8%) and impossible cases for UAE were 2 (0.2%). Retreatment was required in 23 cases (3.3%). Complication events designated by the Society of Interventional Radiology Clinical Practice Guidelines occurred in 31 cases (4.4%): 1 case showed permanent adverse sequelae, 4 cases required major therapy with a prolonged hospitalization (>48 h) and 10 patients required therapy with minor hospitalization (<48 h). The remaining 16 cases required nominal or no therapy. Follow-up of patients was performed for 599 cases for an average period of 1.2±0.9 years, ranging between 1 month and 6 years. Pregnancy after UAE was observed in 12 cases/15 cycles. Thus, the findings indicate that UAE using gelatin sponge prepared by the pumping method applied to outpatients is a safe and useful treatment procedure.

## Introduction

Surgical treatment of uterine leiomyoma is frequently performed in gynecology. Uterine leiomyoma is a benign tumor, which is sometimes treated with drugs instead of surgery (1–3). However, drugs cannot treat leiomyoma completely. Surgical treatment is often the treatment of choice when patients suffer from dysmenorrhea, abdominal mass and secondary symptoms, such as urinary difficulty, constipation and thrombosis, due to increase in leiomyoma size. In surgical treatment, a laparoscopic operation with less stress is performed more often than an abdominal operation (4). However when the size of the leiomyoma is large, generally a laparoscopic operation is not chosen. When submucosal leiomyoma protrudes into the intrauterine cavity, transcervical resection is performed under a hysteroscope to eliminate the leiomyoma (5).

Other than medical and surgical therapies, embolization is also used to treat myoma. A method of treating myoma by embolizing the feeding artery was reported in 1995 (6), in which embolizing material is injected through a catheter (7) inserted into the uterine artery. Since then, the materials and methods for embolization have improved and this method is used world-wide as the third treatment method.

Our clinic has employed uterine artery embolization (UAE) using gelatin sponge particles prepared by the pumping method (7–9) since April 2001, a procedure that has been carried out in the outpatient department without requiring hospitalization. This study reports UAE performed for a large cohort (700 patients between 2001 and 2009) with gelatin sponge particles prepared using the pumping method. We report the results, adverse effects and complications.

## Materials and methods

### Leiomyoma cases

The UAE reported in this study is a routine medical treatment. An Institutional Review Board does not exist in our clinic. The principles of the Declaration of Helsinki were followed.

Patient cases treated by UAE included: i) cases with symptomatic leiomyoma, ii) cases for whom abdominal operation was risky or adverse cases after receiving myomectomy or cervical myoma, iii) cases rejecting surgical operation and iv) cases wishing to become pregnant in the future who selected UAE after sufficient explanation of informed consent.

A giant leiomyoma reaching the umbilicus was generally contraindicated for UAE, due to the following disadvantages associated with a giant leiomyoma: occurrence of numerous incomplete embolization cases, insufficient or a lower reduction rate of the node of embolized leiomyoma and numerous cases of infection after UAE.

The classification of cases according to occurrence sites of leiomyoma was: subserous leiomyoma in 271 cases (38.7%), intramural leiomyoma in 63 cases (9.0%), submucosal myoma in 147 cases (21.0%), cervical leiomyoma in 15 cases (2.1%) and unknown in 204 cases (29.1%). The classification of cases according to the number of leiomyoma was: single leiomyoma in 318 cases (45.4%) and multiple leiomyoma in 382 cases (54.6%). The average age of subjects was 41.0±5.6 years (range, 23–72), and the average age of leiomyoma onset was 36.7±6.5 years (range, 21–54). Table I shows the detailed age distribution of the leiomyoma cases.

The main symptoms of leiomyoma were: hypermenorrhea and anemia in 358 cases (51.1%), dysmenorrhea in 42 cases (6.0%), abdominal mass in 179 cases (25.6%), infertility in 10 cases (1.4%) and other symptoms in 111 cases (15.9%). Regarding the presence and absence of marriage, unmarried cases were 212 (30.3%), married cases were 453 (64.7%) and divorced cases were 35 (5.0%). Parity of subjects was zero in 215 cases (30.7%), once in 85 cases (12.1%), twice in 132 cases (18.9%) and three to four times in 24 cases (3.4%). Patients who underwent previous leiomyomectomy were 70 cases (10%).

### Procedure of UAE in our clinic

Whether treatment of leiomyoma was necessary was judged from results of internal examination, ultrasonic examination and magnetic resonance imaging (MRI) (GE Healthcare Japan, Co., Ltd., Tokyo). When treatment was judged to be necessary, patients were provided with an explanation of the advantages and disadvantages of treatments by drugs, surgical operation, UAE or focused ultrasound surgery, and patients were permitted to select one of these treatments. When patients selected UAE, preoperative examination was carried out and the advantages and disadvantages of UAE were explained again. UAE was confirmed when a signed letter of consent was received from the patients.

The 700 patients were asked to visit our clinic at twelve o’clock on the day of operation, and the following steps were taken: gowning, setting up drip infusion, setting up apparatus for continued subcutaneous injection into the abdomen, and, if necessary, cutting excess pubic hair. UAE was performed by a radiologist with a gynecologist as an assistant. A catheter was inserted into the approach site at the right femoral artery under X-ray guidance (7). Pelvic arteriography was performed using a catheter (Silux Co., Ltd., Kawaguchi). Then, for uterine arteriography, the tip of a catheter was introduced into the uterine artery. The depth of the inserted catheter was usually not allowed to go beyond the descending or horizontal uterine artery. When the uterine arteriography was finished, embolizing material was carefully injected. Gelatin sponge particles (Astellas Pharma, Inc., Tokyo), prepared by crushing gelatin sponge (Astellas Pharma, Inc.) by the pumping method just prior to surgery, were used as embolizing material (8). Gelatin sponge was crushed within two rock-type 10-ml syringes (Terumo Corp., Tokyo) connected with a T-shape stopcock (Terumo Corp.) by liquefying a contrast agent (Fuji Pharma Co., Ltd., Toyama) and gelatin sponge. Pumping was repeated 5 or 6 times. A detailed procedure for the preparation of gelatin sponge particles is as follows (9). Gelatin sponge being absorbed with water was compressed into one of the syringes and the air was evacuated. The other syringe containing the contrast agent was connected via the T-shape stopcock with the gelatin-containing syringe. The gelatin sponge was crushed by alternately pumping the two syringes. Gelatin was slowly injected at a free-flow rate, and embolization was stopped at the endpoint when the procedure was completed at the horizontal artery or root of the ascending artery branch, while checking that the blood flow gradually disappeared at the uterine artery periphery. If the ovary artery of the same side appeared during the embolizing operation, embolization was stopped at this point as a general rule. For cases wishing to become pregnant in the future, a relatively small amount of embolizing material was injected.

For pain control, two tablets of indomethacin (Dainippon Sumitomo Pharma Co., Ltd., Osaka) were administered orally as premedication prior to UAE. Just prior to UAE, a butterfly needle was subcutaneously set up firmly at the epigastric area and 0.5 ml/min of Stadol^®^ (Bristol-Myers Co., Ltd., Tokyo) was continuously infused with a DIB^®^ catheter (Mitsuya Medical, Co., Ltd.). Just after UAE finished, 30 mg Pentagin™ (Astellas Pharma, Inc.) and 25 mg Atarax™ (Pfizer Japan, Inc., Tokyo) were injected intravenously, and if pain occurred thereafter Pentagin was additionally injected intramuscularly. If other symptoms, such as nausea, cramp of the lower extremities and hypertension, occurred, supportive measures were employed.

Just after UAE finished, analgesics and antibiotics were injected intravenously. Thereafter, vital checks were performed in the recovery room adjacent to the operation room. Two to three hours after UAE, pressure hemostasis at the approach site was removed and patients were moved in a wheelchair to the room of a neighboring hotel. On the day after the stay in the hotel, patients returned to the clinic in a wheelchair and the continuous-infusion apparatus was removed. The general status and puncture wound were checked, and then drip infusion was initiated by which the blood concentration of analgesics decreased, allowing for recovery of the state of consciousness and general conditions to be promoted. When the patients’ consciousness cleared and they were able to walk without assistance, patients were allowed to go home, usually at about noon in most cases.

For the follow-up after UAE, patients were asked to visit the hospital 1 week, 1, 3, 6 months and 1 year after UAE: the results of the treatment were evaluated by observing the transitional change of symptoms and by gynecologic, ultrasonographic and MRI tests. The presence or absence of myoma embolization was examined and, if any was detected, the degree of leiomyoma embolization was evaluated and the size of leiomyoma was measured. Therapy evaluation using contrast MRI to check whether the blood stream flow was conducted 1 week after UAE. Infertility treatment was permitted 6 months after UAE.

During the follow-up period, among the 700 patients, 101 patients did not return to our clinic 1 month after UAE. For the 599 patients who returned to our clinic 1 month after UAE or later, the average period of follow-up was 1.2±0.9 years, ranging between 1 month and 6 years. At 1 year after UAE, 331 patients returned to our clinic and 5 patients visited 5 years after UAE.

## Results

### Total results

A total of 700 patients received UAE between April 2001 and March 2009, and the cumulative results (at least one follow-up was performed in a year) were as follows: the average time required for UAE was 15.1±11.7 min. (0.5–145 min) and that for radiation fluoroscopy was 7.8±5.6 min (2.1–61.2 min).

The operation was stopped for 2 cases due to technical reasons: in one case catheter operation was impossible due to arterial sclerosis and in the other case catheter insertion was impossible due to a small uterine artery.

Of the remaining 698 UAE cases, 680 (97.2%) cases were effective and 18 (2.6%) cases were ineffective.

Among the 680 cases in which UAE was effective, complete embolization judged by MRI was observed in 663 cases (97.5%) and incomplete embolization was observed in 17 cases (2.5%). Among the 17 incomplete cases, cases in which leiomyoma recurred were 5 (29.4%).

The prognosis of the 17 incomplete cases was: abdominal surgery in 6 cases, retrial of UAE in 7 cases (1 case received both these treatments) and 5 cases received no treatment or were unknown. Leiomyoma was completely embolized in all 7 retrial cases of UAE cases.

The 31 (4.4%) patients complaining of complications were classified according to the Society of Interventional Radiology Classification System for Complications by Outcome (10) (Table II). Among minor complications, 3 patients did not require therapy and 13 patients received nominal therapy with no consequence. Among major complications, 10 patients required therapy with minor hospitalization (<48 h), 4 patients required major therapy and an unplanned increase in level of care and prolonged hospitalization (>48 h), 1 patient had permanent adverse sequelae, although no deaths occurred. Intrauterine infection (11 cases), deterioration of ovary function (5 cases) and Asherman’s syndrome (5 cases) were included in these hospitalized patients.

Degenerated cervical leiomyoma after UAE was observed in 15 of 700 cases (2.1%). Expulsion was observed in 4 of these 15 cases (26.6%) on days 6, 7, 18 and 21 after UAE.

When the ineffective cases of UAE and recurrence were examined in 140 cases of giant leiomyoma and in 560 cases of non-giant leiomyoma, ineffective cases were higher in giant leiomoyama cases (11 cases, 7.8%) compared to non-giant cases (7 cases, 1.2%). Recurrence occurred in 3 cases (2.1%) of giant leiomyoma cases and in 2 cases (0.3%) of non-giant leiomyoma cases, but was a little higher in the giant leiomyoma cases.

Among 10 cases receiving UAE twice, repeated surgery was due to ineffectiveness (5 cases) and recurrence (5 cases). Leiomyoma was found to have steadily decreased in size in 8 cases following the second UAE, while no effect was observed in 1 case and follow-up was impossible in 1 case.

A total of 295 cases wished to become pregnant, including 23 cases under infertility treatment. At the time of writing, the patients who became pregnant after UAE comprised 19 cases and 22 cycle with an average age of 33.4±3.1 years (range, 30–38). The time between UAE and pregnancy was between 2 months and 5.2 years with the cycle of becoming pregnant after infertility treatment being 6 cycles and the remaining 16 cycles being achieved in a natural cycle. The prognosis of pregnancy included 3 cases of natural abortion, and among 15 cases of delivery, 8 cases were transvaginal and 7 cases were cesarean delivery. No cases of preterm delivery, placenta accreta and uterine rupture occurred.

## Discussion

This study on UAE performed for a large cohort of patients confirmed the advantage of gelatin-sponge particles prepared using the pumping method. In our clinic, liquefied gelatin sponge (8) is used as an embolizing material for UAE, as in other Japanese hospitals. Most Japanese doctors have not used polyvinyl alcohol, which is usually used in the United States of America and Europe, mainly because this foreign material is not anabolic (degradable) and is expensive.

Gelatin sponge is processed using either the cutting method to cut the sponge into 1 mm particles, or the pumping method (11). We have used the pumping method since first adopting this procedure and continue to do so at present. The pumping method is much easier to perform than the cutting method. We repeated pumping 5 or 6 times, and the distribution of the size of gelatin particles prepared by 5 repetitions of pumping was relatively wide compared with the size distribution of the gelatin powder according to Mori *et al* (9): the size distribution of gelatin powder is <1.6 mm, and that of pumping method-prepared particles is <6.6 mm being not much different from that of the cutting method. Makuuchi *et al* (12) noted that the rate of bile duct necrosis is high when gelatin powder is used.

Compared with the rate of efficacy of UAE carried out in the United States of America and Europe (13–16), the rate of efficacy of our clinic was markedly high at 97.2%. Gabriel-Cox *et al* (15) reported that 18% of women underwent hysterectomy after UAE. Spies *et al* (16) reported a 25% chance of failure of symptom control or recurrence after UAE. UAE requires only 15 min operating time compared with an average of 1–2 h generally required for abdominal laparotomy, and therefore UAE can be said to be a less invasive treatment for patients. The exact mechanism underlying why our UAE method using gelatin particles prepared by the pumping method provided favorable results remains to be clarified.

We emphasize that the majority of patients were satisfied with UAE treatment. Our preliminary questionnaire investigation on a total of 158 patients performed 1 year after UAE showed that 77 (48.7%) were entirely satisfied and 69 (43.6%) were satisfied, while 10 (6.3%) felt not too bad and 2 (1.2%) were dissatisfied. One of the two dissatisfied patients felt no improvement because she had no symptoms from the beginning, and the other patient of age 47 was not satisfied because she reached menopause.

The Committee on Gynecological Practice, the American College of Obstetricians and Gynecologists (ACOG) considers the procedure of UAE to be investigational or relatively contraindicated in women wishing to retain fertility (13). This recommendation is assumed to be issued because ovarian dysfunction after UAE and uterine amenorrhea are considered. Pregnancies after uterine artery embolization have higher rates of preterm delivery and malpresentation than those after laparoscopic myomectomy (17). Firouznia *et al* (18) reported that among 102 women who underwent bilateral UAE using polyvinyl alcohol, 23 (22.5%) wished to become pregnant: 14 of the 23 (61%) became pregnant and nine were nulliparous. These authors concluded that randomized comparisons with myomectomy should be performed to determine whether uterine artery embolization is safe for women who wish to retain their fertility.

In our clinic, UAE is carried out only if patients select it after the above-mentioned disadvantage is explained sufficiently. Numerous patients selected UAE, even after considering the advantages and disadvantages of conventional leiomyoma treatment by abdominal surgery (especially leiomyomectomy) and of UAE for leiomyoma treatment to reduce or remove leiomyoma. In our clinic, a milder embolizing procedure was attempted for patients wishing to become pregnant. Of note, injection of embolizing material is stopped when the ovarian artery is observed during UAE. Pregnancy has been established in 19 cases/22 cycles thus far. The 295 cases who still wished to become pregnant were various and included ‘seeking pregnancy at all means’, ‘unmarried cases’ and ‘seeking pregnancy if possible’; therefore, the reason could not be determined. The process to become pregnant involved pregnancy after infertility treatment at 6 cycles, and the remaining 16 cycles were pregnancy in a natural cycle.

## Figures and Tables

**Table I:** Age distribution of subjects.

Age (years)	No. of cases
<20	0
20–24	3
25–29	24
30–34	82
35–39	179
40–44	265
45–49	131
>50	16
Total	700

**Table II:** Classification of complications of 31 patients.

	Classification of complications	No. of patients
Minor complications		
A	No therapy, no consequence	3
B	Nominal therapy, no consequence	13
Major complications		
C	Require therapy, hospitalization (<48 h)	10
D	Require major therapy, hospitalization (>48 h)	4
E	Permanent adverse sequelae	1
F	Death	0
